# Reduced Glutathione Protects Subcellular Compartments From Pb-Induced ROS Injury in Leaves and Roots of Upland Cotton (*Gossypium hirsutum* L.)

**DOI:** 10.3389/fpls.2020.00412

**Published:** 2020-04-15

**Authors:** Mumtaz Khan, Samrana Samrana, Yi Zhang, Zaffar Malik, Muhammad Daud Khan, Shuijin Zhu

**Affiliations:** ^1^Department of Agronomy, Institute of Crop Sciences, College of Agriculture and Biotechnology, Zhejiang University, Hangzhou, China; ^2^Department of Environmental Sciences, Faculty of Engineering, Gomal University, Dera Ismail Khan, Pakistan; ^3^Department of Biotechnology and Genetic Engineering, Kohat University of Science and Technology, Kohat, Pakistan; ^4^Department of Soil Sciences, University College of Agriculture & Environmental Sciences, The Islamia University of Bahawalpur, Bahawalpur, Pakistan

**Keywords:** heavy metals stress, cell morphology, reactive oxygen species, antioxidant enzymes, chloroplast, mitochondria

## Abstract

Heavy metals-based changes in the plants and their alleviation through eco-friendly agents including reduced glutathione (GSH) have been widely studied. In the present experiment, we tested the alleviatory role of reduced glutathione (GSH) in seedlings of upland cotton cultivar, TM-1 under lead (Pb) toxicity. Plants were grown in the Hoagland solution containing Pb (0 μM), Pb (500 μM), GSH (50 μM), and GSH + Pb (50 μM + 500 μM). Lead exposure exacerbated hydrogen peroxide (H_2_O_2_) and hydroxyl radical (OH^•^) levels, induced lipid peroxidation (MDA), and decreased the activities of catalase (CAT) and ascorbate peroxidase (APX) in the terminal and median leaves of 28-days old cotton seedlings stressed for 10 days. However, in the primary and secondary roots, CAT activity was increased but APX decreased. Similarly, peroxidase (POD) and superoxide dismutase (SOD) activities were enhanced in the median leaves but a declining trend was observed in the terminal leaves, primary roots and secondary roots. Glutathione reductase (GR) activity, ascorbic acid (AsA) contents and GSH concentrations were increased in all parts except AsA in the median leaves. Transmission electron micrographs of Pb-treated plants exhibited deformed cell wall and cell membrane, disfigured chloroplasts and irregularly shaped mitochondria in the terminal and median leaves. Further, cell membrane, mitochondria, nucleus and other cell organelles in root cells were severely affected by the Pb. Thus their identification was little bit difficult through ultramicroscopy. External GSH stabilized leaf and root ultramorphology by stabilizing cell membranes, stimulating formation of multivesicular body vesicles, and by maintaining structural integrity of other organelles. Evidently, GSH played major alleviatory role against Pb toxicity in upland cotton.

## Introduction

Environmental lead (Pb) has increased 1000 fold in the past three centuries, mainly due to huge industrial demands and less recycling, causing its accumulation in the plants and animals (ATSDR, 2007; Rahman and Singh, 2019). Its uptake causes physiological and morphological changes in plant’s organs including roots and leaves (Jiang et al., 2014b; Khan et al., 2016). Moreover, biomolecules such as proteins, nucleic acids, lipids and hormones are also affected by the Pb (Pourrut et al., 2011; Lanier et al., 2019). During metal stress, excess reactive oxygen species (ROS) such as superoxide anion (^•^O^–^), hydrogen peroxide (H_2_O_2_), hydroxyl ion (OH^•^) and malondialdehyde (MDA) are produced which cause oxidative damage to cell membranes and biomolecules (Das and Roychoudhury, 2014; Berni et al., 2019). Plants respond to excess ROS by inducing enzymatic and non-enzymatic antioxidants like superoxide dismutase (SOD), catalase (CAT), ascorbate peroxidase (APX), peroxidase (POD), glutathione reductase (GR), ascorbic acid (AsA) and reduced glutathione (GSH) that work synergistically to cope with metal toxicity (Ali et al., 2014a; Berni et al., 2019).

Among antioxidants, SODs constitute cell’s first line defense against ROS (Mishra and Sharma, 2019). Based on metal cofactor and protein folding, SODs have been grouped into Fe-SOD, Mn-SOD and Cu-Zn-SOD (Szõllõsi, 2014). They dismutate highly reactive ^•^O^–^, produced at the sites of electron transport chain, to form less toxic H_2_O_2_ and O_2_. Both, CAT and APX neutralize H_2_O_2_, however; former is more effective scavenger due its higher affinity for H_2_O_2_ (Cuypers et al., 2016). Peroxidases are abundantly present in the plant cells and use H_2_O_2_ as a substrate to produce H_2_O and O_2_. Glutathione reductase reduces oxidized glutathione (GSSG) back into GSH to maintain balanced levels of the later in cells (Lu, 2013). Ascorbic acid scavenges ROS through glutathione-ascorbate pathway and serves as a cofactor for various enzymes (Hasanuzzaman et al., 2019).

Reduced glutathione, found in the most life forms, is bestowed with myriad of cell functions (Hausladen and Alscher, 2017). It is biosynthesized as a tripeptide of glutamate, cysteine and glycine in two ATP-dependent steps by glutamate cysteine ligase (“GSH1”) and glutathione synthetase (“GSH2”) enzymes. In addition to its role as precursor for phytochelatins (PCs), GSH plays roles in redox signaling, ion homeostasis and sulfur assimilations. Moreover, it maintains catalytic and regulatory thiol groups in the reduced state (Zechmann, 2014). In earlier research work, GSH has alleviated oxidative stress, induced by range of pollutants, in several plant species (Sobrino-Plata et al., 2014; Daud et al., 2016; Adams et al., 2020). Besides this, GSH promotes PCs synthesis in a transpeptidation reaction catalyzed by phytochelatin synthase (Grill et al., 1987). Being functionally analogous to metallothioneins, PCs bind to heavy metals (HMs) through thiol group (Cobbett, 2000). Further, in GSH redox cycle, it reduces H_2_O_2_ and lipid peroxides via reactions catalyzed by GSH peroxidase. During this process, GSH is oxidized to GSSG, which can be reversed to GSH by GR, depending on NADPH, thereby keeping optimum GSH pools for proper cell functioning. However, HMs adversely affect GSH concentrations in the plants (Eroglu et al., 2015), and that external feeding of GSH may help alleviate oxidative stress in upland cotton.

In previous experiment, we have studied the alleviatory effect of exogenous GSH on photosynthesis and chloroplast morphology in Pb-stressed cotton seedlings (Khan et al., 2016). However, how GSH alleviates Pb stress in different leaves and roots of cotton needs a detailed study. Keeping in view of this fact, we designed current experiment to observe Pb-triggered biochemical and morphological changes in terminal and median leaves, and primary and secondary roots of upland cotton, and subsequent recovery through reduced glutathione application.

## Materials and Methods

### Growth Conditions and Plant Sectioning

Upland cotton variety TM-1 was selected for this study which is a standard genetic line and comparatively a heavy metal tolerant variety. Its seeds were obtained from Cotton Germplasm Lab, Zhejiang University, Hangzhou, China, and grown hydroponically according to Khan et al. (2016). Briefly, sterilized upland cotton seeds were grown in growth chamber adjusted to 16/8 h day/night, 60/80% humidity, 45 μE m^–2^ s^–1^ irradiance, and 350 μM M^–1^ CO_2_ concentrations. Uniformly selected 28-days old seedlings were categorized into four groups; (1) control (0 μM Pb), (2) 500 μM Pb as Pb (NO_3_)_2_, (3) 50 μM GSH, and (4) 50 μM GSH + 500 μM Pb. These treatment levels were selected based on earlier findings from screening tests done in our lab and cotton’s seedling threshold level. After 10 days of stress period, harvested plants were separated into terminal leaves (fully expanded upper pair of terminal leaves excluding apical region), median leaves (excluding cotyledonary leaves), primary roots and secondary roots. Seedling stage is the best time to study biochemical and morphological changes in leaves and roots of plants. Plant samples were either freshly used for biochemical assays or stored at −80°C till further analyses.

### Sample Extraction for Biochemical Assays

Enzyme extracts (EE) were obtained from plant samples to determine ROS, antioxidant enzymes and AsA content. Briefly, samples were treated with 10 M Na-EDTA to remove unbound Pb, and thoroughly washed with distilled water. Then 8 mL of pre-cooled phosphate buffer solution (PBS, 16.385 g Na_2_HPO_4_.12H_2_O L^–1^ and 0.663 NaH_2_PO_4_.2H_2_O L^–1^), adjusted to pH 6.5, was added to 0.5 g plant sample in a pre-cooled mortar and pestle. Thoroughly homogenized samples were centrifuged at 12000 × *g* for 20 min at 4°C and supernatants were collected in 10 mL tubes, and kept at −30°C till further use.

### Assays for Reactive Oxygen Species

Reactive oxygen species such as H_2_O_2_, OH^•^ and MDA were determined according to Kaur et al. (2015) with slight modifications. Briefly, H_2_O_2_ was determined based on KI oxidation by H_2_O_2_. To 1 mL PBS, 2 mL potassium iodide (KI, 1 M) and 1 mL EE were added and absorbance was recorded at 390 nm. Molar extinction coefficient (ε) of 0.028 μM cm^–1^ was used to calculate H_2_O_2_ contents and expressed as μM g^–1^ fresh weight. For MDA, 1.5 mL EE and 2.5 mL mixture of 5% trichloroacetic acid (TCA) + 5% thiobarbituric acid (TBA) was incubated at 95°C for 15 min and then immediately cooled on ice. After short centrifugation at 4800 × *g*, the absorbance of MDA-TBA adduct was measured at 532 nm and 600 nm. The MDA content was determined using ε = 155 mM cm^–1^ and expressed as μM g^–1^ fresh weight. For OH^•^ determination, the reaction mixture comprised of 0.7 mL EE, 3 mL 0.5% TBA and 1 mL glacial acetic acid. After incubation at 100°C for 30 min, the reaction mixture was immediately cooled at 4°C and absorbance was measured at 532 nm. The OH^•^ concentration in the sample was calculated using extinction coefficient, ε = 155 mM cm^–1^ and values were expressed as μM g^–1^ fresh weight.

### Antioxidant Assays

The activities of CAT and APX were determined according to Ali et al. (2014b). For CAT, the reaction solution contained 0.1 mL of 300 mM H_2_O_2_, 0.1 mL EE and 2.8 mL PBS. The reaction mixture was shaken gently before taking reading at 240 nm. One unit of CAT activity was considered equal to the amount of enzyme required to catalyze one micro mole of H_2_O_2_ per min. For APX, the reaction mixture contained 0.1 mL H_2_O_2_ (300 mM), 0.1 mL EE, 0.1 mL AsA (7.5 mM) and 2.7 mL PBS. The absorbance was measured at 290 nm. One unit of APX activity was defined as the amount of enzyme needed to oxidize one micro mole of ascorbate per minute. The POD and SOD activities were determined according to Mei et al. (2015). For POD, the reaction mixture was comprised of 0.1 mL each of EE, guaiacol (1.5%) and H_2_O_2_ (300 mM), and 2.7 mL PBS. The absorbance was measured at 470 nm. For SOD activity, a reaction solution of 75 μM nitroblue tetrazolium (NBT), 20 μM riboflavin, 100 μM Na-EDTA and 130 mM methionine was prepared. Then, 2.725 mL reaction solution, 0.25 mL ddH_2_O and 0.025 mL EE were mixed and kept under light (4000 Lux) for 20 min, followed by recording absorbance at 560 nm. One unit of SOD activity was equal to the amount of enzyme required to inhibit 50% of NBT reduction. The GR activity was determined as decline in NADPH levels at 390 nm absorbance using extinction coefficient of 6.2 mM cm^–1^ (Jiang and Zhang, 2001).

### Pb and GSH Quantification

Lead contents in leaves and roots of upland cotton were determined according to Khan et al. (2016). Briefly, plant materials were weighed, ashed in muffle furnace and acid digested. Then homogenate was filtered several times to get clear extract for Pb quantification by ICP-MS. Pb contents were expressed as μg g^–1^ fresh weight. For GSH determination, plant samples (0.4 g) were grinded with 4 mL trichloroacetic acid (5% v/v) and the homogenate was centrifuged at 4000 × *g* for 10 min at 4°C to get supernatant. To 2.6 mL NaH_2_PO_4_ (150 mM, pH 7.8), 0.25 mL supernatant was added and shaked. Then, 0.18 mL DNTP, dissolved in phosphate buffer, was added and mixture was kept at 300°C for 5 min. Absorbance was measured at 412 nm and GSH was quantified against standard curve.

### Root Scanning and Transmission Electron Microscopy

Cotton roots were scanned by MIN MAC, STD 1600^+^ root scanner (Regent Instruments) using WinRhizo software for image acquisition. Transmission electron microscopy of terminal and median leaves and primary roots and secondary roots was done according to Khan et al. (2016). Briefly, small leaf and root sections (2–3 mm) were excised by a sharp blade and kept overnight in 3% glutaraldehyde (prepared in 1 M PBS, pH 7.2), and vacuumed several times before fixing in 1% OsO_4_ for 1.5 h. Then graded series of ethanol i.e., 50–100% and absolute acetone were used to dehydrate samples. Samples were then put in 1:1 and 3:1 mixture of absolute acetone and final spur resin for 1 and 3 h, respectively. After, keeping the samples overnight in final spur resin, 100 nm thin samples, cut on a ultramicrotome (Reichert Ultracut E, Germany), were stained with 6% aqueous uranyl acetate and Renold’s lead citrate, and mounted on copper grids. Micrographs were taken on transmission electron microscope model-7650, Hitachi Japan operating at 80 kv.

### Data Analysis

Data obtained from three replications were subjected to ANOVA, using statistical software package, SPSS 16.0 (SPSS Inc., United States). Significant differences among various treatment means were determined at 5% probability by Duncan’s multiple range test. The data were presented as mean ± SD (standard deviation) of three independent biological replicates.

## Results

### Generation of Stress Biomarkers

Data revealed that ROS levels in leaves and roots of upland cotton were elevated upon Pb exposure, followed by reduction through GSH application (Table 1). In control group, leaves generated more H_2_O_2_ than roots. Pb caused a significant percent increase of 52, 46, 41 and 53 in H_2_O_2_ in terminal leaves, median leaves, primary roots and secondary roots respectively as compared with control. GSH alone treatment increased H_2_O_2_ levels in all studied plant parts except secondary roots. GSH + Pb treatment maximally reduced H_2_O_2_ in primary roots than terminal and median leaves, while less reduction was found in secondary roots. Similarly, highest increase (vs. respective controls) in the OH^•^ due to Pb was found in primary roots (98%) and secondary roots (80%) as compared to terminal (67%) and median leaves (60%). The highest decline in OH^•^ was caused by GSH in terminal leaves in the presence of Pb. Although, GSH reduced OH^•^ levels in the terminal and median leaves, its levels were still higher than primary and secondary roots. Notably, after Pb exposure, primary roots faced more lipid peroxidation (MDA) than terminal and median leaves, and secondary roots. GSH effectively controlled lipid peroxidation in all plant sections except median leaves.

**TABLE 1:** Lead (Pb) and reduced glutathione (GSH) mediated hydrogen peroxide (H_2_O_2_), hydroxyl ion (OH^•^) and malondialdehyde (MDA) contents in upland cotton leaves and roots.

**Treatments**	**Terminal leaves**	**Median leaves**	**Primary roots**	**Secondary roots**
**H_2_O_2_ (μM g^–1^ FW)**				
Control	753.6b	65.371.7c	45.262.4b	9.980.6c
500 μM Pb	1134.4a	95.333.3a	64.002.3a	15.241.8a
50 μM GSH	794.8b	78.588.4b	37.871.2c	9.830.8c
50 μM GSH + 500 μM Pb	638.5c	56.898.9c	32.991.8d	12.571.1b
**OH^•^ (μM g^–1^ FW)**				
Control	0.020.001c	0.020.001c	0.030.002c	0.020.004c
500 μM Pb	0.060.002a	0.050.001a	0.120.004a	0.100.007a
50 μM GSH	0.030.001b	0.020.002c	0.020.002c	0.020.001c
50 μM GSH + 500 μM Pb	0.030.001b	0.030.003b	0.060.002b	0.070.011b
**MDA (μM g^–1^ FW)**				
Control	923.9b	766.5bc	70.9b	90.9b
500 μM Pb	1726.3a	1295.6a	141.6a	110.9a
50 μM GSH	724.5d	649.9c	70.9b	70.9c
50 μM GSH + 500 μM Pb	825.3c	877.1b	60.1b	70.9c

*Data presented here was obtained from three independent biological replications. Means possessing same alphabets are not significant at 5% probability as determined by Duncan’s multiple range test. The values are means ± standard deviation (SD).*

### Impact of Pb and GSH on Antioxidants

**FIGURE 1:**
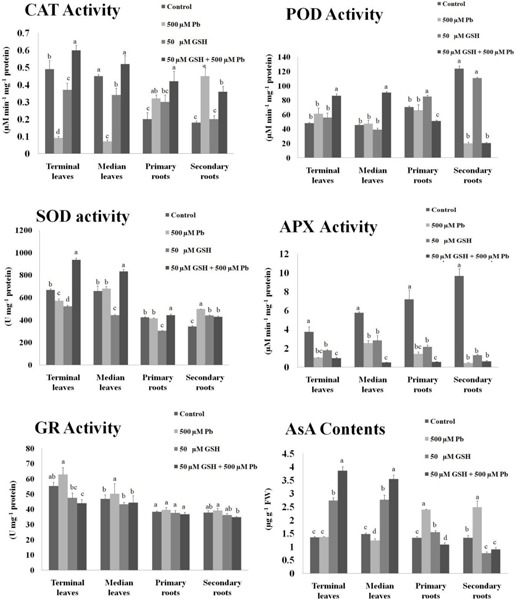
Antioxidant activities in upland cotton under Pb and GSH applications. Response of catalase (CAT), peroxidase (POD), superoxide dismutase (SOD), ascorbate peroxidase (APX), glutathione reductase (GR) and ascorbic acid (AsA) in cotton leaves and roots exposed for 10 days to Pb and GSH in Hoagland solution. Same alphabets on bars represent no significance at 5% probability according to Duncan’s multiple range test. Values are mean ± standard deviation (SD) obtained from three independent biological replicates.

Results demonstrated changes in the activities of antioxidant enzymes upon Pb, and Pb + GSH exposures (Figure 1). The CAT activity in Pb alone treatment was declined in the terminal (0.09 μM/min/mg protein) and median leaves (0.07 μM/min/mg protein), but was elevated in plants subjected to GSH + Pb treatment. The POD activity was boosted in the Pb + GSH treatment in the terminal (85.86 μM/min/mg protein) and median leaves (90.56 μM/min/mg protein), but a declining trend was observed in the primary (51.01 μM/min/mg protein) and secondary roots (20.34 μM/min/mg protein). The highest POD activity was observed in the control group. A differential response was observed for the SOD activity in various treatments. The SOD activity was declined in the terminal leaves (570 U/mg protein) and primary roots (415 U/mg protein) but secondary roots (497 U/mg protein) demonstrated an increase in the Pb group as compared with respective control. However, all parts showed increase in the SOD activity in GSH + Pb group, with highest seen in the terminal leaves. The APX activity was declined in the Pb and GSH + Pb groups in all plant sections as compared with control. However, maximum APX activity (9.65 μM/min/mg protein) was recorded in the secondary roots of control group and lowest (0.48 μM/min/mg protein) in the median leaves of GSH + Pb group. Pb stress caused increase in GR activity in the cotton seedlings with peak levels reported in the terminal leaves (62.84 U/mg protein) and least in the secondary roots (36.23 U/mg protein). The highest GR activity in the GSH + Pb treated plants was observed in the median leaves (44.28 U/mg protein) and lowest in the secondary roots (34.79 U/mg protein). The AsA contents were declined in the terminal and median leaves but increased in both primary and secondary roots under Pb stress. GSH alone increased AsA contents in all parts except secondary roots (0.76 μg/g FW). However, GSH with Pb combination significantly increased AsA contents in the terminal and median leaves but the response was meager in the primary and secondary roots.

### Pb Accumulation and GSH Content

**FIGURE 2:**
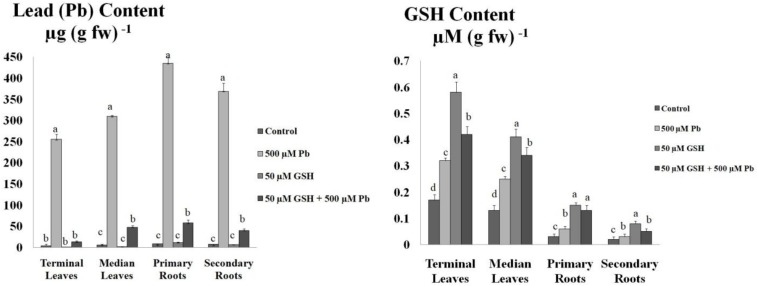
Lead (Pb) and GSH concentration in upland cotton leaves and roots. Upland cotton seedlings exposed to Pb (500 μM) and GSH (50 μM) alone and in combination showing Pb uptake and GSH concentration in terminal leaves and median leaves, and primary roots and secondary roots. Error bars on columns show standard deviation among the values. Chart data are means of three independent biological replications, analyzed through one way analysis of variance (ANOVA) and significant difference determined by Duncan’s multiple range test at 0.05% probability level.

Results revealed that maximum Pb uptake was observed in the primary roots followed by secondary roots (Figure 2). Further, Pb was significantly translocated to the upper foliation but its levels in the median leaves were higher than terminal leaves. GSH application significantly reduced Pb uptake in the leaves and root system of upland cotton. After GSH exposure, least Pb accumulation was found in the terminal leaves while highest in the primary roots.

### Leaf Morphology and Microscopy Under Pb and GSH Treatments

**FIGURE 3:**
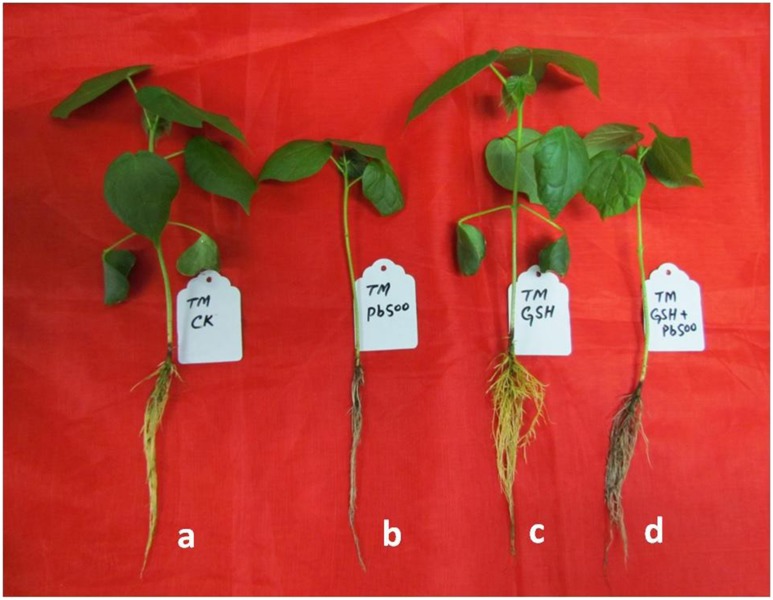
Visual impact of Pb and GSH on upland cotton seedlings. Photographs of upland cotton seedlings (variety TM-1) representing control **(a)**, and those treated with 500 μM Pb **(b)**, 50 μM GSH **(c)** and 500 μM Pb + 50 μM GSH **(d)**.

**FIGURE 4:**
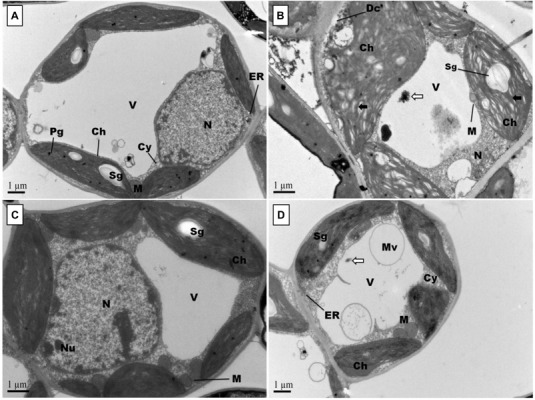
Effects of Pb and GSH on ultrastructure of upland cotton terminal leaves. Transmission electron micrographs represent leaf cell morphology of control **(A)**, 500 μM Pb **(B)**, 50 μM GSH **(C)** and 500 μM Pb + 50 μM GSH **(D)** in cotton seedlings. In control cells **(A)**, cytoplasm (Cy) contained large central vacuole (V), nucleus (N) and well developed chloroplasts (Ch) with starch grains (Sg) and plastoglobuli (Pg). Rounded mitochondria (M) and endoplasmic reticulum (ER) can also be detected. After Pb exposure **(B)**, chloroplasts were highly dilated and granal stacks, represented by black arrow (→), were broken. Similarly, electron dense particles and cell debris, represented by white arrow (⟸), was accumulated in the vacuole. GSH-treated leaf cells **(C)** possessed large chloroplasts, nucleus, nucleolus (Nu), and number of mitochondria were located in between chloroplasts. In GSH + Pb group **(D)**, chloroplasts were dilated but still intact. Moreover, large vacuole with multivesicular structure (Mv), endoplasmic reticulum and nucleus with nucleoli (Nu) were present also.

**FIGURE 5:**
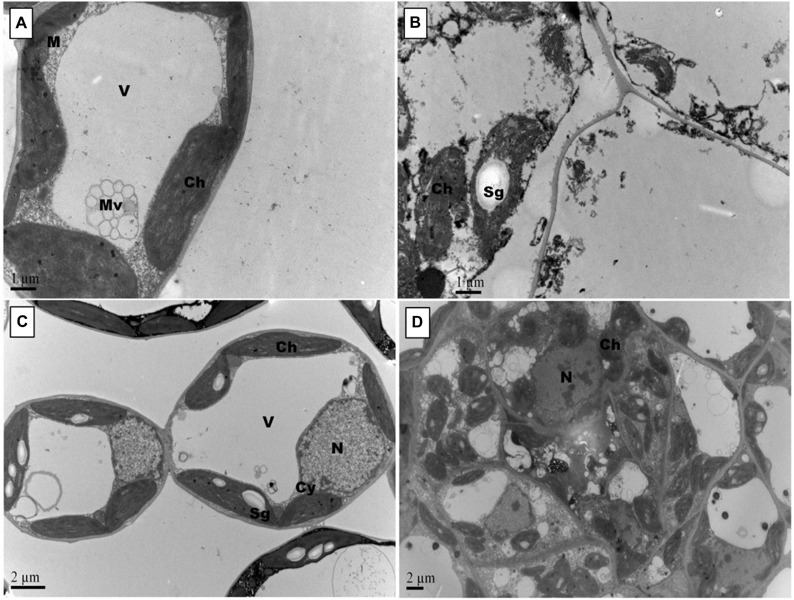
Effects of Pb and GSH on ultrastructure of upland cotton median leaves Transmission electron micrographs represent leaf cell morphology of control **(A)**, 500 μM Pb **(B)**, 50 μM GSH **(C)** and 500 μM Pb + 50 μM GSH **(D)** in cotton seedlings. Control cells **(A)**, contained large central vacuole (V), well developed chloroplasts and rounded mitochondria (M). After Pb exposure **(B)**, chloroplasts were highly dilated and plasma membrane detached from cell walls. GSH-treated leaf cells **(C)** demonstrated large chloroplasts and clearly located nucleus. In GSH + Pb group **(D)**, chloroplasts were dilated but increased in number.

Representative photographs clearly showed the adverse effects of Pb and the alleviating impact of GSH on the Pb-treated cotton seedlings (Figure 3). As a whole, there were few small leaves on the Pb-treated cotton seedlings having black roots while larger leaves and denser roots were observed in the GSH treatments. Distinct differences were also observed in the transmission electron micrographs (TEMs) of Pb- and GSH-treated cotton terminal and median leaves (Figures 4, 5). In control plants, mesophyll cells in both types of leaves were occupied by a bulky central vacuole which pressed plasma membrane against the cell wall. Cytoplasm possessed several adjoining chloroplasts, mitochondria, a large nucleus with scattered chromatin network, endoplasmic reticulum (ER) and ribosomes. Well-developed chloroplasts with elliptical starch grains were observed near cell wall which contained integrated granal stacks and lamellae. Spherical mitochondria, found at chloroplast junctions, were enriched with cristae and electron dense granules. However, clear nucleoli were invisible in the median leaves. In the Pb group, swollen chloroplasts were displaced from the peripheral cell wall boundary. The granal stacking and intergranal lamellae were disfigured. Similarly, mitochondria lost round shape and thread-like structures, probably cell debris and electron dense molecules, were accumulated in bulky vacuoles. The adverse effects of Pb were more pronounced in the median leaves as compared with terminal leaves. In GSH-treated plants, terminal and median leaves contained large well-developed chloroplasts, crowded with grana stacks and lamellae. Nucleus possessed highly condensed chromatin network and visible nucleolus. However, contrary to the control and Pb treatments, bulky vacuole was squeezed due to the presence of “mega” size chloroplasts and dilation of the nucleus in terminal leaves. Moreover, several mitochondria were assembled in the terminal leaves at chloroplast intersections, recognizable by their typical round shape. In the GSH + Pb group, dilated chloroplasts were filled with several starch grains in both terminal and median leaves. In the central vacuole, multivesicular structures were seen instead of dense electron particles or thread-like structures. Moreover, mitochondria were swollen and created gaps between two chloroplasts. But these gaps were larger in the median leaves as compared with terminal leaves.

### Root Morphology and Microscopy Under Pb and GSH Treatments

**FIGURE 6:**
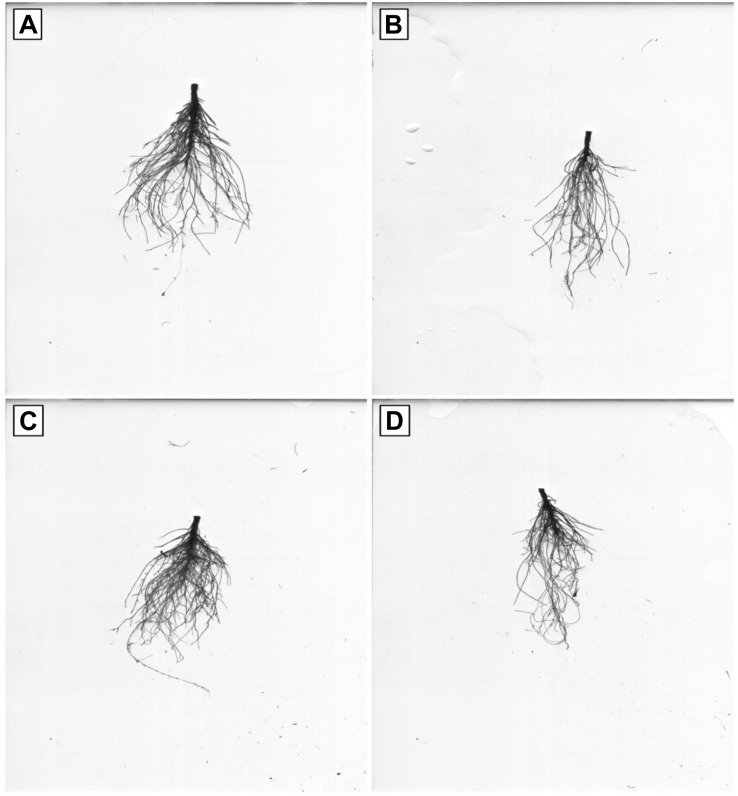
Effects of Pb and GSH on growth and length of primary and secondary roots. Scanned roots represent control **(A)**, cotton roots treated with 500 μM Pb **(B)**, cotton roots treated with 50 μM GSH **(C)** and root of plant treated with combined application of 500 μM Pb and 50 μM GSH **(D)**.

**FIGURE 7:**
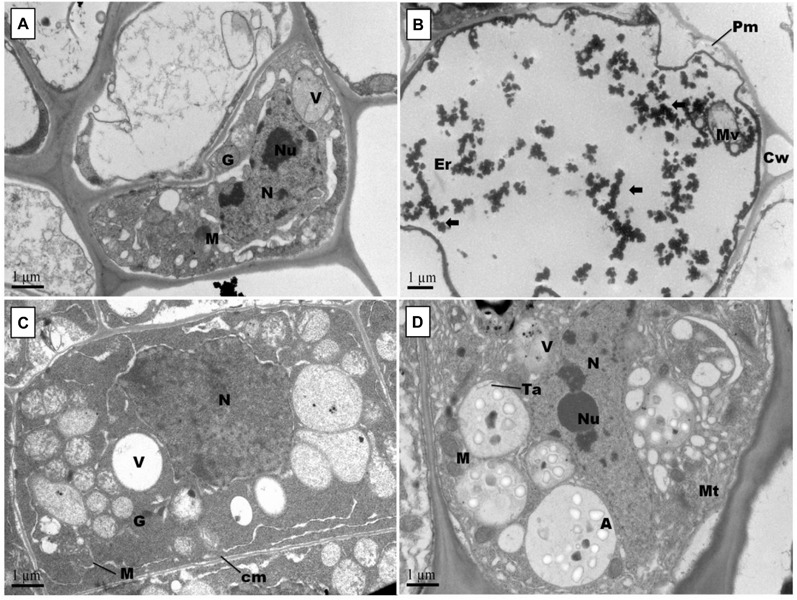
Effect of Pb and GSH on primary root ultrastructure in upland cotton Transmission electron micrographs represent root tips cell morphology of control **(A)**, 500 μM Pb **(B)**, 50 μM GSH **(C)** and 500 μM Pb + 50 μM GSH **(D)** in cotton seedlings. Control cells **(A)** posses cytoplasm embedded with nucleus (N) containing nucleoli (Ne), vacuole (V), Golgi vesicles (G) and several mitochondria (M). In Pb-treated cells **(B)**, plasma membrane (Pm) was broken and cytoplasm severely damaged. Electron dense particles (⟸) were scattered throughout the cell. GSH **(C)** induced formation of vacuoles, mitochondria and multivesicular structures. In GSH + Pb **(D)**, large multivesicular structures contained amyloplasts (A), thylakoids (Ta) and microtubules (Mt).

**FIGURE 8:**
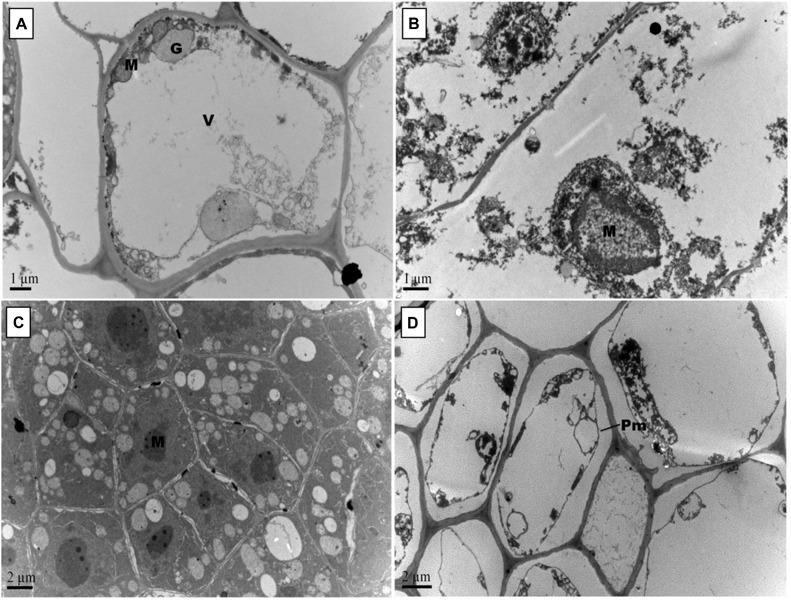
Effect of Pb and GSH on secondary root ultrastructure in upland cotton. Transmission electron micrographs show secondary roots cell morphology of control **(A)**, 500 μM Pb **(B)**, 50 μM GSH **(C)** and 500 μM Pb + 50 μM GSH **(D)** in cotton seedlings. Control cells **(A)** possessed large central vacuole (V), Golgi vesicles (G) and mitochondria (M). In Pb-treated cells **(B)**, cell morphology was severely altered. GSH-treated roots **(C)** had developed cell organelles and formation of several multivesicular vesicles. In GSH + Pb **(D)**, plasma membrane (Pm) was detached from cell wall in almost every cell.

Root scanning revealed that Pb stress reduced density of cotton roots; however, GSH increased root density in primary and secondary roots (Figure 6). Like leaves, Pb changed the orientation of subcellular organelles in primary and secondary roots of cotton (Figures 7, 8). Under control conditions, TEMs displayed plasma membrane pressed to thick cell walls. Moreover, the cytoplasm contained multiple cell organelles like mitochondria, Golgi vesicles, vacuole and centrally located large nucleus with nucleoli in primary roots. TEMs of Pb-treated roots exhibited a devastating ultramorphology, with collapsed membrane-bound compartments in both primary and secondary roots. The plasma membrane was ruptured and seen scattered between cytoplasm and cell wall. The cytoplasm itself was highly dilated, probably due to increased permeability of cell wall and plasma membrane, and covered almost entire cell lumen. The cytoplasm contained electron-dense particles, presumably disintegrated cell organelles and Pb particles. Only few endoplasmic reticulum and multivesicular body vesicles were noticed in the root micrographs. No mitochondria or nucleus were clearly located inside the cell. The adverse effects were more pronounced in the secondary roots as compared to primary roots. In the GSH-treated group, primary root cell micrographs showed cell organelles embedded in the cell lumen (Figure 5C). But plasma membrane was discontinues due to expansion caused by multivesicular body vesicles and vacuoles. Multivesicular body vesicles were mainly located longitudinally at opposite poles and possessed dense particles. Contrary to leaf micrographs, multiple small vacuoles were found, which contained cell debris. Centrally located nucleus was marked with dense chromatin material and less mitochondria. In the GSH + Pb group, plasma membrane appeared intact and pressed to the thick cell wall in both primary and secondary roots. Moreover, cytoplasm had large numbers of clear and denser multivesicular body vesicles. The central vacuole was filled with cell debris and, probably, with Pb particles. Multiple mitochondria were located near cell wall and nucleus in primary roots but unclear in secondary roots. Nucleus was stretched across whole cell length, with nucleoli being located in the center. Endoplasmic reticulum and dictyosomes were seen distributed near cell wall. Further, spherical amyloplasts contained thylakoids, and abundant microtubules were found in the cells of primary roots. However, there was less growth recovery in secondary roots, and clear identification of cell organelles was difficult.

## Discussion

Previous research has shown that cotton can accumulate significant amounts of heavy metals including Pb in seeds, leaves and roots, thereby bringing physiological, metabolic and ultrastructural changes (Daud et al., 2009; Khan et al., 2016). However, study about the role of GSH in modulating antioxidant enzymes and alleviating the ultrastructural-based alterations under Pb-toxicity in terminal and median leaves, and primary and secondary roots of upland cotton seedlings is scarce in the available literature. Leaves are the primary organs of photosynthesis and transpiration in plants while roots absorb essential elements and water from the soil. So, alterations in their ultramorphology and biochemistry may cause growth retardation in plants.

ROS such as H_2_O_2_, OH^•^ and MDA are stress indicators (Cuypers et al., 2016). They affect enzymes, proteins, nucleic acids and intracellular organelles; however, intensity and pathway of ROS generation is dependent on the cell organelles and stress conditions (Thyrring et al., 2015). Mostly, ROS are produced at active sites in the cells such as cell membranes, mitochondria, endoplasmic reticulum and peroxisomes; however, they migrate through plasma membrane and can be found anywhere in the cell. In current study, higher H_2_O_2_ generation and lipid peroxidation (MDA) was observed in the cotton’s terminal and median leaves as compared with primary and secondary roots (Table 1). This is because of more active sites of ROS generation in leaves than roots. Moreover, GSH neutralized ROS levels in cotton median and terminal leaves and primary and secondary roots. Basically, GSH neutralizes ROS by donating H^+^ and keeps protein cysteine in active reduced form by offering e^–^ (Lu, 2013). The ability of GSH as reducing agent is dependent on the GSH/GSSG ratio and total glutathione concentration, which are negatively affected by stress conditions. Moreover, higher GSH-mediated ROS control was observed in leaves as compared to roots in this study. It may be due to GSH-mediated restricted translocation of Pb to the foliar regions (Nakamura et al., 2013) and enhanced GSH synthesis in the leaves (Frendo et al., 1999). Further, two most important steps of glutathione-mediated xenobiotics detoxification identified in the plants are chemical/toxicant transformation and compartmentation (Coleman et al., 1997; Singh et al., 2016). In the first instance, electrophilic xenobiotics, which have high affinity toward making covalent bonds with nucleophilic sites, are transformed into less toxic forms by conjugating with glutathione in cytosol, thereby increasing their hydrophilicity and decreasing biological half life. In the second step, conjugates are transported to the vacuoles or across plasma membrane by ATP-dependent transporters, like one present in the tonoplast. Plants have the ability to produce two types of Cys-containing metal binding legends i.e., metallothioneins and phytochelatins, which are functionally analogous (Shukla et al., 2016). Role of glutathione in phytochelatin synthesis is well documented as it serves as its precursor and increase plant tolerance to metal stress (Vezza et al., 2019). Under heavy metal stress, phytochelatin synthase, a key enzyme in phytochelatin synthesis, is activated which causes increased formation of phytochelatins. Phytochelatins make complexes with heavy metals including Pb and subsequently sequestered in vacuoles where more complex aggregation occurs (Sharma et al., 2016).

In earlier work, changes in CAT, POD, SOD, APX, and GR under Pb have been reported in plants (Pourrut et al., 2011; Ali et al., 2014a). However, variation in enzyme activities is dependent on Pb concentrations, time of exposure, plant species examined, etc. In heavy metal stress, GSH plays dual role; as an antioxidant metabolite and as a precursor for PCs (Hausladen and Alscher, 2017). The antioxidant property of GSH is dependent on its reduced cysteine moiety, which is oxidized when GSH reduces target molecules (Pompella et al., 2003). Results showed modulation of CAT, POD, SOD, and APX activities in both terminal and median leaves, and primary and secondary roots of upland cotton (Figure 1). Previously, GSH has alleviated cadmium stress in upland cotton (Daud et al., 2016), chromium stress in rice (Cao et al., 2013) and cesium stress in Arabidopsis (Adams et al., 2020); mainly by regulating antioxidants. Furthermore, GR activity was enhanced in the terminal and median leaves, but remained nearly unchanged in the groups treated with Pb alone and in combination with GSH. Previously, it was also observed that GSH levels were increased during stress conditions, but GR activity remained unchanged, pointing to the GSH *de novo* synthesis (Liu et al., 2015). In current study, Pb contents and GSH concentrations in terminal and median leaves and primary and secondary roots were also measured (Figure 2). Results revealed that GSH significantly reduced Pb uptake in both leaves and roots. This may be due to increase in the internal GSH concentrations caused by the external application as previously reported by Adams et al. (2020). Membrane stability, phytochelatin synthesis and compartmentalization of Pb, mediated by GSH may be contributing factors for Pb detoxification and restricted upward translocation to the aerial parts (Adams et al., 2020).

Changes in cell wall ultrastructure of terminal and median leaves (Figures 4, 5) and primary and secondary roots (Figures 7, 8) under Pb stress and some re-establishment of intact structures by GSH were observed in the current study. Pb alters cell wall morphology by increasing K^+^ influx from the cell, and turnover of cell wall-bound Ca^2+^ and Mg^2+^, creating nutrient deficiency and altering other cell structures and functions that depend on the intactness of these elements (Sharma and Dubey, 2005; Ali et al., 2014c). Moreover, Pb-inspired-ROS generation may accelerate lipid peroxidation by converting unsaturated fatty acids into saturated ones, causing damage to the plasma membrane (Thyrring et al., 2015). In earlier research work, GSH has played role in stabilizing plasma membrane under stress conditions (Zechmann, 2014). Conformational changes in chloroplasts upon Pb exposure were observed in terminal and median leaves of upland cotton. It is well established that chloroplasts have the inbuilt capacity to generate ROS; however, it is fortified under stress conditions (Khan et al., 2019). In addition, chloroplast proteome possesses reactive cysteines which are required for the catalytic functions of proteins and work under the control of glutaredoxin and thioredoxin systems (Zaffagnini et al., 2012; Couturier et al., 2013). These reactive cysteines are sensitive to ROS-triggered oxidation. Further, heavy metals replace Mg^2+^ ions in the chlorophyll and form heavy metal-chlorophyll complex (HM-*Chl*), for example cadmium (Cd)-*chl* complex (Küpper et al., 2006). Chlorophylls with heavy metal-complex face conformational changes due to light exposure and acidic nature of thylakoids. Moreover, it is suggested that chlorophyll degradation is not only caused by Pb-*Chl* complex but by generation of O^∙−^ also, which is triggered by this complex. Further, Pb^2+^ being analogous to Mg^2+^ may replace the later in chlorophylls, causing further deterioration in the chloroplast ultrastructure. A recent study, conducted on *Lemna trisulca* L. has reported rearrangement of chloroplast as an avoidance strategy to Pb stress (Samardakiewicz et al., 2015) which is at par with our findings. Moreover, GSH promotes glutathionylation, a process of protein posttranslational modifications via disulphide bond, using H_2_O_2_ as stimulant in chloroplasts (Zaffagnini et al., 2012), thus playing role in stabilizing chloroplast ultrastructure.

In this study, mitochondria were seen deformed both in terminal and median leaves, and primary and secondary roots of upland cotton under Pb exposure. These changes may be attributed to excess ROS generation, as mitochondria release free electrons in electron transport chain to form O^∙−^ and other ROSs (Dourmap et al., 2019). As a first line defense, SODs neutralize O^∙−^ in mitochondria, that’s why SOD activity was increased in GSH + Pb group. Moreover, O^∙−^ may cause H_2_O_2_ production, which can be neutralized by CAT, but the later is present in peroxisomes only, providing strong evidence for H_2_O_2_ neutralization through GSH redox system (Lu, 2013), and alleviating Pb stress. In GSH redox system, H_2_O_2_ is reduced to H_2_O by GPX mainly using GSH as a substrate (Ribas et al., 2014). Further, NADPH-dependent GR reduces GSSG, mainly found in the mitochondria, to GSH and may be the second important enzyme to regulate the function and structure of mitochondria.

Pb also altered cell nuclear matrix in the terminal and median leaves, and primary and secondary roots of upland cotton in current study. Previous research has shown some release of nuclear proteins such as nucleophosmin, nucleolin and fibrillarin from nucleolus to the cytoplasm in *Allium cepa* L. root tips cells upon exposure to Pb (Jiang et al., 2014a). These nuclear proteins play important roles in protein formation and ribosomal biogenesis. Moreover, Pb inhibits DNA synthesis, which may lead to reduced and impaired mitosis and cell division (Patra et al., 2004). We observed nuclear stabilization due to GSH application, both in leaves and roots. Importantly, nucleus contains GSH levels twice than cytosol, which are closely paralleled by DNA synthesis in early stages of cell proliferation (Noctor et al., 2012). Even under stress conditions, GSH supply to the nucleus remains uninterrupted (Zechmann, 2014). Thus, it is suggested that a continuous supply of GSH to the nucleus from cytoplasm might have maintained the nuclear stability in leaves and roots of upland cotton under Pb stress.

## Conclusion

Lead is an unavoidable toxic heavy metal in the agro-ecosystems. In this multi-organ study, Pb increased ROS generation and decreased the activities of CAT, POD, SOD, APX, and SOD but increased GR activity in the terminal and median leaves, and primary and secondary roots of upland cotton. External GSH neutralized excess ROS levels, optimized activities of antioxidant enzymes and reduced Pb uptake in leaves and roots. However, maximum decline in the Pb uptake and peak GSH contents were observed in the terminal leaves. Moreover, Pb deteriorated ultrastructural configuration in both type of leaves and root system by affecting cell wall, plasma membrane, chloroplasts, mitochondria, and nucleus. GSH application maintained better cell morphology in the terminal leaves and primary roots as compared to other parts, mainly by regulating antioxidant machinery and restricted uptake of Pb. However, how DNA is regulated under external GSH supply in cotton leaves and roots in the presence of Pb needs further investigation.

## Data Availability Statement

The datasets generated for this study are available on request to the corresponding author.

## Author Contributions

MK, SS, and YZ equally contributed to the lab work. ZM and MDK prepared figures, tables and conducted transmission electron microscopy. SZ acquired funding, supervised this research work and critically revised the manuscript.

## Conflict of Interest

The authors declare that the research was conducted in the absence of any commercial or financial relationships that could be construed as a potential conflict of interest. The reviewer MF declared a shared affiliation, with no collaboration, with several of the authors ZM, SS, YZ, and SZ to the handling Editor at the time of review.
